# Novel Bioactive Glass/Graphene Oxide-Coated Surgical Sutures for Soft Tissue Regeneration

**DOI:** 10.1021/acsomega.3c00978

**Published:** 2023-06-08

**Authors:** Kerim Emre Öksüz, Begüm Kurt, Zeynep Deniz Şahin İnan, Ceylan Hepokur

**Affiliations:** † Department of Metallurgical and Materials Engineering, Faculty of Engineering, Sivas Cumhuriyet University, Sivas 58140, Türkiye; ‡ Department of Gynecology and Obstetrics, Faculty of Medicine Hospital, Sivas Cumhuriyet University, Sivas 58140, Türkiye; § Department of Histology-Embryology, Faculty of Medicine, Sivas Cumhuriyet University, Sivas 58140, Türkiye; ∥ Department of Biochemistry, Faculty of Pharmacy, Sivas Cumhuriyet University, Sivas 58140, Türkiye

## Abstract

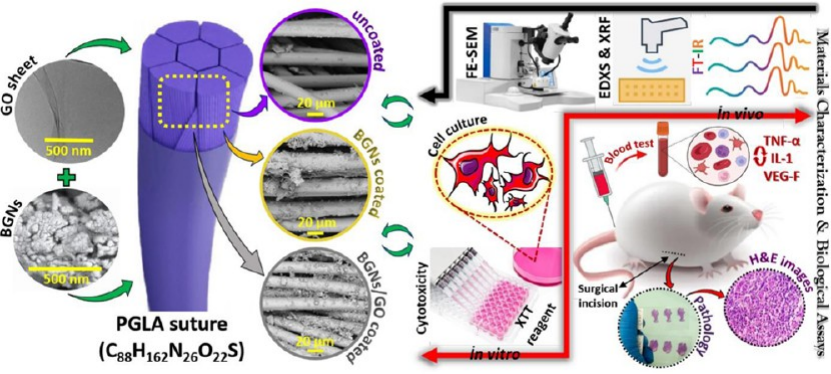

The combination of a commercially available PGLA (poly[glycolide-*co*-l-lactide]), 90:10% suture material with bioactive bioglass nanopowders (BGNs) and graphene oxide (GO)-doped BGNs offers new opportunities for the clinical application of biomaterials in soft tissue engineering. In the present experimental work, we demonstrate that GO-doped melt-derived BGNs were synthesized via the sol–gel process. After that, novel GO-doped and undoped BGNs were used to coat resorbable PGLA surgical sutures, thereby imparting bioactivity, biocompatibility, and accelerated wound healing properties to the sutures. Stable and homogeneous coatings on the surface of the sutures were achieved using an optimized vacuum sol deposition method. The phase composition, morphology, elemental characteristics, and chemical structure of uncoated and BGNs- and BGNs/GO-coated suture samples were characterized using Fourier transform infrared spectroscopy, field emission scanning electron microscopy, associated with elemental analysis, and knot performance test. In addition, in vitro bioactivity tests, biochemical tests, and in vivo tests were performed to examine the role of BGNs and GO on the biological and histopathological properties of the coated suture samples. The results indicated that the formation of BGNs and GO was enhanced significantly on the suture surface, which allowed for enhanced fibroblast attachment, migration, and proliferation and promoted the secretion of the angiogenic growth factor to speed up wound healing. These results confirmed the biocompatibility of BGNs- and BGNs/GO-coated suture samples and the positive effect of BGNs on the behavior of L929 fibroblast cells and also showed for the first time the possibility that cells can adhere and proliferate on the BGNs/GO-coated suture samples, especially in an in vivo environment. Resorbable surgical sutures with bioactive coatings, such as those prepared herein, can be an attractive biomaterial not only for hard tissue engineering but also for clinical applications in soft tissue engineering.

## Introduction

Sutures are still the most often used method of wound closure in surgical applications because they are widely accessible and efficiently provide the required mechanical support during the wound healing period.^1^ A wide variety of suture materials are commercially available, and the surgeon can select sutures with a variety of properties to find the best fit for the specific needs of the existing wound. Considerations for selecting a suitable suture for wound closure and healing include suture strength, tissue holding power, absorbability, infection risk, and the inflammatory reaction associated with the suture material. Infections occurring up to 30 days after surgery (or up to one year following surgery in patients receiving implants) and affecting either the incision or the deep tissue at the operation site are referred to as surgical site infections. Despite advancements in prevention, surgical site inflammations continue to be a serious clinical concern since they are associated with considerable mortality and morbidity.^2,3^ For many years, researchers have researched synthetic bioabsorbable surgical sutures based on polyglycolic acid (PGA), polylactic acid (PLA), and poly(glycolide-*co*-lactide) (PGLA) for resolving clinical problems.^4^ Novel sutures, described as bioactive sutures, are being created to potentially improve wound healing by releasing collagen components into the damaged or wounded area. Bioactive components may also help to accelerate wound healing by transferring various cellular lines from the suture to the wounded sites.^1,5^ There are various requirements for the creation of clinically acceptable bioactive sutures, which necessitates extensive study in the evolution of these products. Regarded as ideal, the material should be inexpensive, and the advantage of the sutures should be provable (especially with in vivo tests). The more complicated the structure of a biomaterial to be obtained and the more chemical components are added into the structure, the greater the risk of antigenicity and the risk of allergic reactions.^6^ Polymeric biostructures appear to be an excellent choice for mimicking the natural structure of soft tissues; nevertheless, various additives are frequently added to the polymeric matrix to increase biological characteristics and hence accelerate wound healing.^7,8^ Because of their unique features, bioceramics (BGs, bioactive glasses (45S5 Bioglass, S53P4,13-93B1,13-93B3)), carbon nanostructures (graphene (G), graphene oxide (GO), carbon dots (CDs), carbon nanotubes (CNTs)), and hydroxyapatite (HAp, β-TCP, α-TCP)^9−11^ have been considered as potential materials for soft tissue repair and regeneration procedures.

The limited results^12−15^ of in vivo animal studies in soft tissues on BGNs and carbon-based biomaterials in the literature, researchers observed increased wettability and improved cell adhesion and development after incorporating BGNs into polymeric scaffolds.^16^ Therewithal, these limited studies have shown that the key advantages of bioceramics particles for soft tissue healing applications are that they promote cell growth and proliferation, induce angiogenesis, and have antimicrobial and anticancer properties.

Carbon-based nanomaterials (CBNs), like bioceramic-based materials, have also shown excellent potential in soft tissue regeneration. The potential of carbon-based structures (CNTs, G) has been reported in repairing wide gaps in injured nerves.^17,18^ As an example of CBNs, the abundant oxygen groups (carboxyl, hydroxyl, epoxy groups) in GO facilitate interfacial contact with polymeric matrices and ceramics, resulting in increased mechanical strength. GO can also promote osteogenic differentiation, angiogenesis, and hydroxyapatite mineralization, which increase calcium fixation.^19^ Therefore, there remains a need to investigate whether composite bioactive surgical sutures can be effective in the wound healing process for soft tissues, as previous studies failed to provide sufficient evidence. To further investigate this matter, in the present work, composite suture materials were fabricated for the first time, combining commercially available resorbable PGLA sutures with GO-doped BGN powder using a vacuum-assisted sol deposition (VSD) technique. The behavior of coated surgical sutures of different compositions (uncoated suture, coated BGNs suture, and coated GO-doped BGNs suture) under in vitro conditions as the cell attachment and cell viability of the bioactive suture was determined using L929 cells. The cell proliferation and wound healing of the repair sites were analyzed by biochemical and in vivo tests and confirmed in order to determine if they have positive impacts on the wound healing process. We report here the influence of BGNs and BGNs/GO on soft tissue healing by comparing the physical and microstructural properties of the coated sutures to the control group where standard sutures without coating are used. Although the potential application of bioglass in soft tissue engineering has been discussed in the literature,^20^ to the authors’ knowledge, this is the first study that quantitatively evaluates the effect of the composition of BGNs and BGNs/GO on the surgical suture with clinical application.

## Results and Discussion

**Figure 1:**
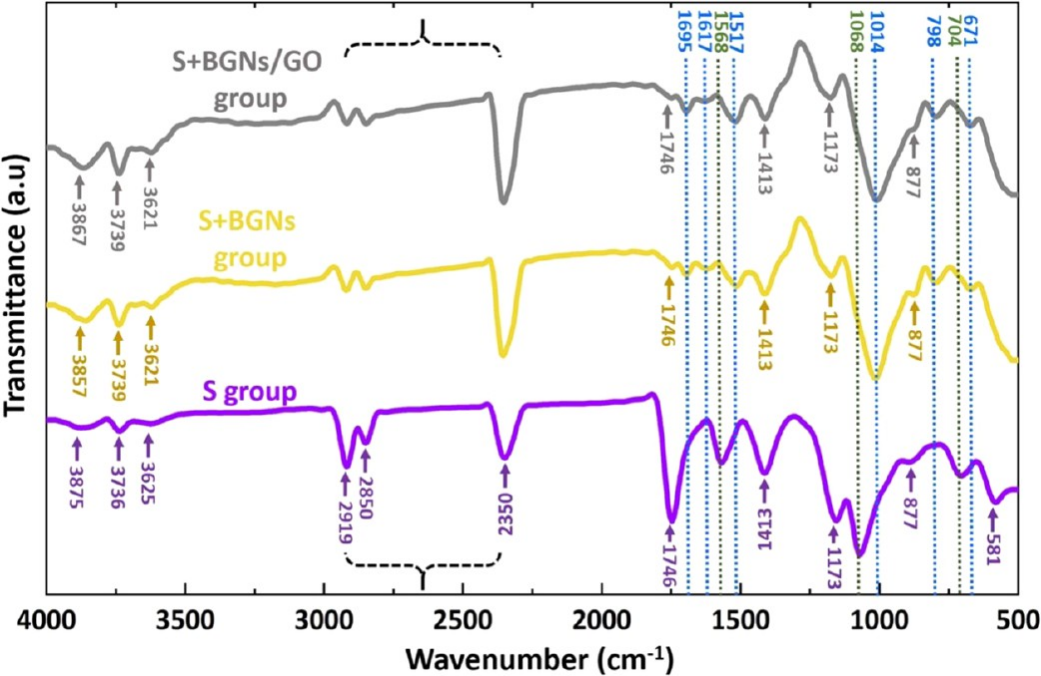
FTIR spectra for the surfaces of S group, S+BGNs group, and S+BGNs/GO group sutures.

Structural assessment and suture surface charge analysis were performed with Fourier transform infrared (FTIR) spectroscopy for wavenumbers in the range of 400–4000 cm^–1^ in the transmittance mode. The FTIR spectra of BGNs- and BGNs/GO-coated suture samples and uncoated reference suture sample are presented in Figure 1, and detailed band positions and more assignments of the suture samples are listed in Table 1.

**Table 1:** Major IR Absorption Bands and Their Assignments in the FTIR Spectrum of Suture Samples

wavenumbers (cm^–1^)			
S group	S+BGNs group	S+BGNs/GO group	band assignments
3875	3857	3867	O**–**H stretch
3736	3739	3739	O**–**H stretch
3625	3621	3621	O**–**H stretch
2919	2919	2919	asymmetric **–**CH_2_**–**, symmetric **–**CH_3_ and **–**CH_2_**–** stretching vibrations
2850	2850	2850	asymmetric **–**CH_2_**–**, symmetric **–**CH_3_ and **–**CH_2_**–** stretching vibrations
2350	2350	2350	O=C=O stretching vibrations
1746	1746	1746	C=O stretching vibrations
	1695	1695	O**–**H bending vibrations
	1617	1617	O**–**H bending vibrations
1568			asymmetric stretching vibrations of (COOH) coordinated with Ca^2+^
	1517	1517	C**–**O asymmetric stretching
1413	1413	1413	asymmetric C**–**H bending
1173	1173	1173	C**–**O**–**C stretching vibrations
1068			C**–**O**–**C stretching vibrations
	1014	1014	Si**–**O**–**Si symmetric and asymmetric stretching
877	877	877	C**–**C=O stretching
	798	798	Si**–**O**–**Si symmetric and asymmetric stretching
704			bending vibration of long-chain (CH_2_)_n_ of Ca^2+^
	671	671	bending vibration of O**–**P**–**O
581			weak bending vibration of long-chain (CH_2_)_n_ of Ca^2+^

Signals corresponding to the vibration of water molecules reacted with the suture composite samples and the −OH functional groups are observed in the region between 3875 and 3621 cm^–1^.^21,22^ In the case of the BGNs/GO combination, only a shift with respect to BGNs is observed due to the incorporation of GO, which contributes with an additional concentration of the −OH groups inherent in GO.^23^ These −OH groups in GO have interacted with BGNs causing a shift of the observed peak to a slightly higher energy region. The main peak associated with the C–H group between 2919–2350 cm^–1^ is strongly observed in the uncoated suture, but its intensity decreases with the addition of BGNs and GO; this decrease has been associated in the literature with a surface degradation of PGLA^24^ during the process of functionalization. The observation of a peak related to the presence of CO_2_ at 2350 cm^–1^ is related to the high sensitivity of PGLA to surface adsorption of ambient CO_2_ molecules.^25^ Besides, it has been observed that the intensity of this peak increases with the incorporation of BGNs, which is related to the morphology of BGNs that promotes the reaction with CO_2_ molecules in the solvent for the reason that BGNs can actively react with CO_2_ during mixing in aqueous solvents.^26^ Likewise, the electrostatic interactions of GO slightly increase the peak intensity due to the tendency of GO to attract CO_2_ molecules in its structure.^27^ Peaks associated with PGLA were observed at 1746, 1413, 1173, and 877 cm^–1^. The intensities of these peaks decreased with the incorporation of BGNs, and this effect was slightly accentuated with the incorporation of GO. These results are related to the sensitivity of these functional groups in PGLA to the electronegative disruption caused by BGNs and GO on the surface of the polymer, which modifies the electronic cloud of the functional group through a van der Waals repulsion phenomenon.^28^ Peaks related to the vibrations of coordinated Ca^+^ ions in calcium stearate are observed at 1568, 704, and 581 cm^–1^ in the uncoated sutures due to a remnant coating from the wire-drawing process of the material. These peaks are not observed in the other combinations as they are overlapped by the incorporation of BGNs and GO.^26^ Another band characteristic of C–O functional groups in the uncoated suture is noticed at 1068 cm^–1^, but this signal has been degraded in BGNs and BGNs/GO sutures, suggesting a high interaction between BGNs and GO with the surface of the suture. The bands at 1014 and 798 cm^–1^ are associated with strong vibrations of the SiO_2_ functional groups in the BGNs. Only a slight broadening is observed with the incorporation of GO, which suggests a strong coupling between the BGNs and GO.^20^ Signals associated with PO_4_^3–^ of the BGNs are detected, which are sensitive to this wavenumber.^29^

**Figure 2:**
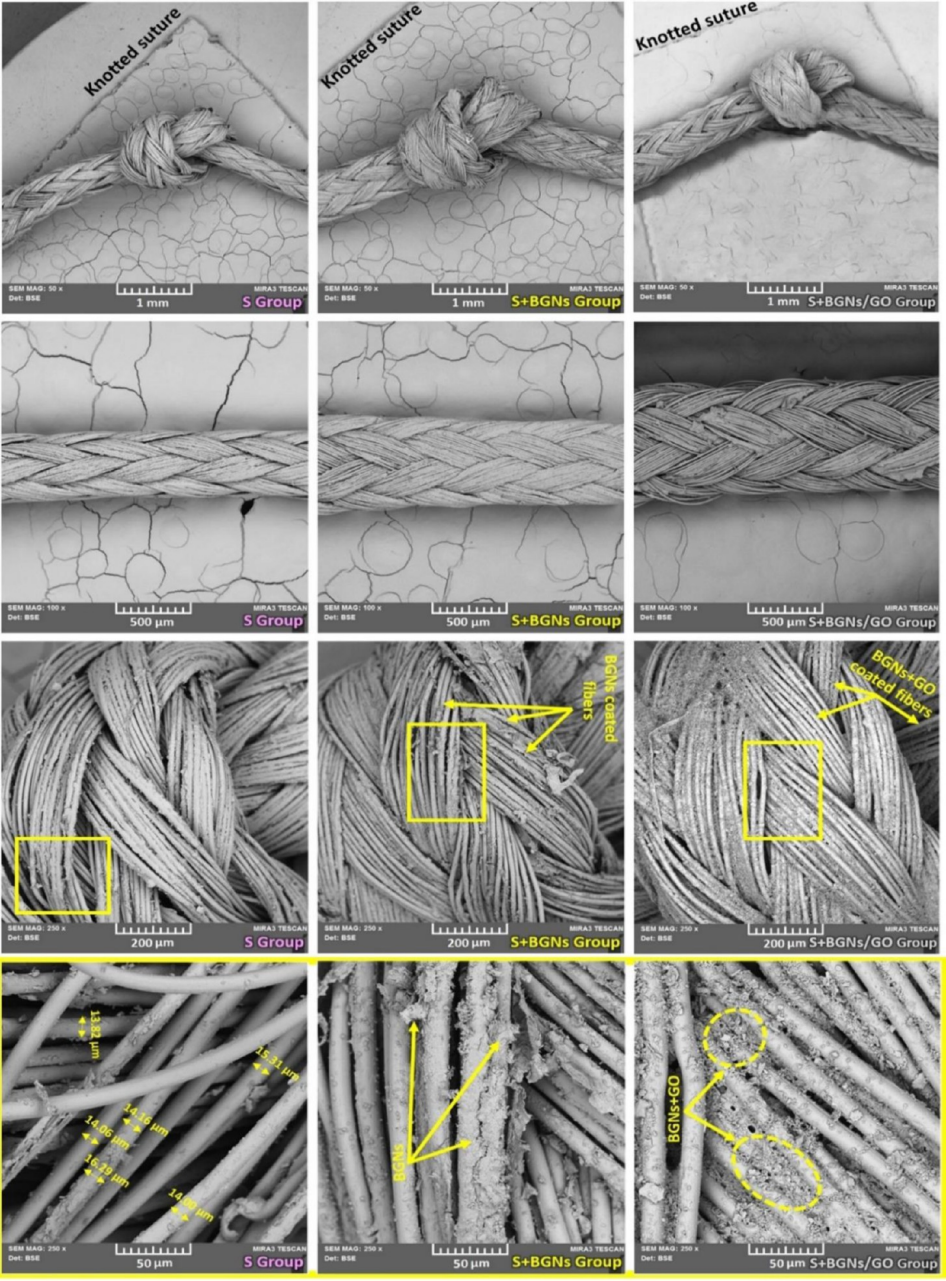
FE-SEM images of the suture composite surfaces, depicting their morphology at different magnifications. The FE-SEM images provide information about the topography, texture, and composition of the surface. In this case, the FE-SEM images illustrate the structural features and surface characteristics of the suture composites, which are important for their mechanical performance and biological properties. The different magnifications used in the images enable a more comprehensive understanding of the surface morphology and the interactions between the different components of the composite.

**Figure 3:**
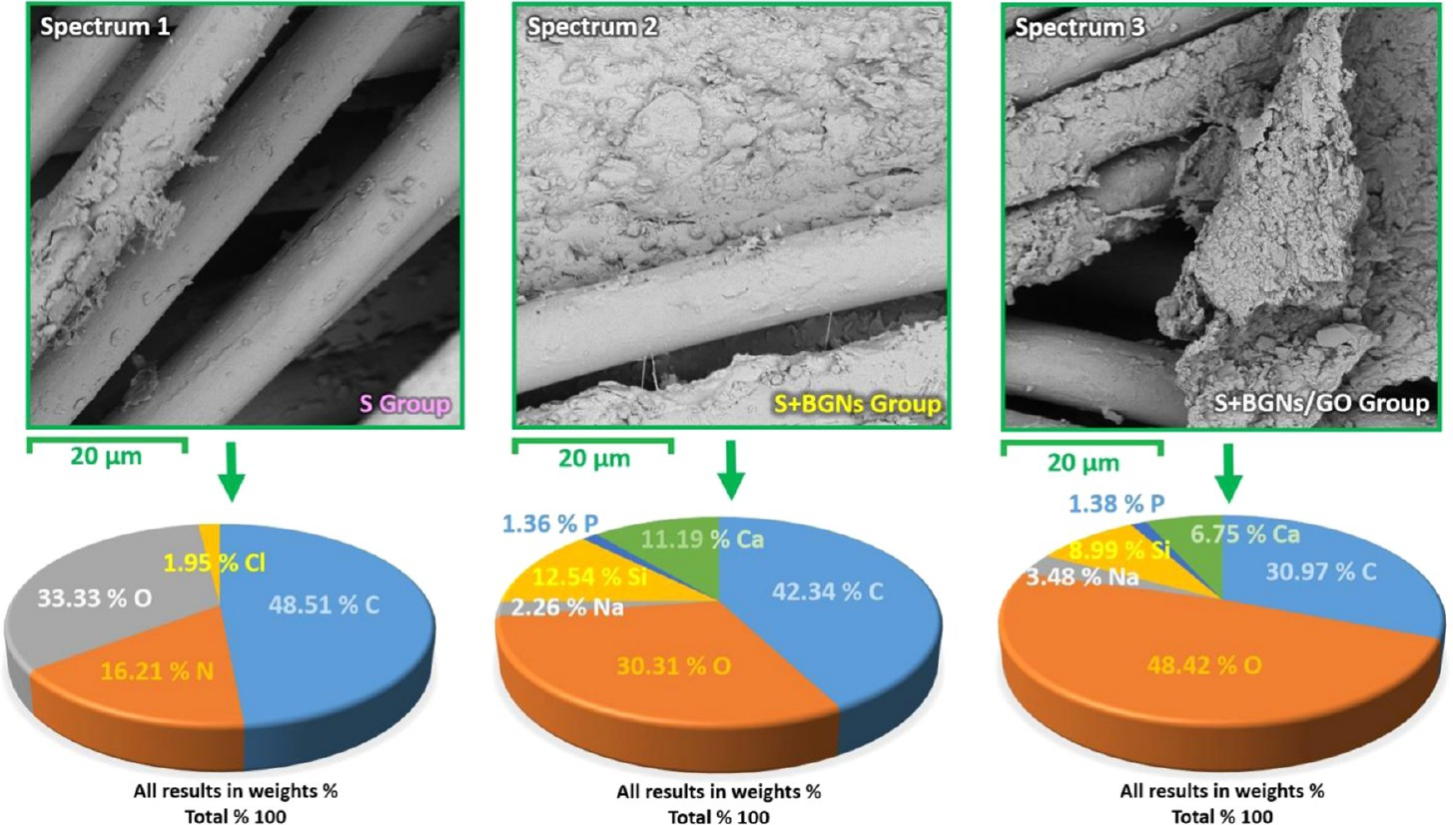
EDX spectra obtained from the FE-SEM images of the surfaces of both as-received and coated suture samples. The spectra obtained from the as-received and coated suture samples provide information about the elements present on the surface and their distribution. The comparison of the spectra can help assess the success of the coating process and the changes in the elemental composition of the suture surface due to the coating.

Visual inspection and FE-SEM analysis of the BGNs and BGNs+GO adhered to the surface of the suture samples were used to undertake qualitative evaluations of the morphology and uniformity of the coatings. Figures 2 and 3 show FE-SEM micrographs and elemental analysis using the EDX spectrum of as-received and BGNs- and BGNs+GO-coated suture samples, respectively. BGNs resulted in more uniform, stable, repeatable, and adherent coatings for suture samples. Coating with BGNs and BGNs+GO with optimal parameters increased the thickness of the coatings in a controllable manner, produced flexible and stable coatings after drying, and decreased the likelihood of microcrack development. S+BGNs and S+BGNs/GO group fibers were slightly rougher than the S group fibers, which might be attributable to the BGNs and BGNs+GO coating on the suture surface. BGNs and BGNs+GO were detected to be scattered on the fibers of coated suture groups. Together with FE-SEM, EDX and FTIR analysis of the coated suture composites demonstrated the presence of BGNs and GO. The average diameter of fiber for each suture group was determined by ImageJ analysis software, and the mean values are reported. Uniform, cylindrical, and stable fibers with a diameter of around ∼20.35 ± 0.14 μm and ∼18.30 μm ± 0.21 were achieved in the S+BGNs and S+BGNs/GO groups, respectively. On the other hand, the fiber diameters of the uncoated sutures were achieved as ∼14.6 ± 0.11 μm for the S group. The adhesion strength of BGNs and GO particles to the coated suture surfaces could not be quantitatively evaluated. FE-SEM was also used to qualitatively determine that the coated sutures retained most of their coating after knotting. The microscopic images show the stability of the coatings, which remained almost intact after surgery. Less exfoliation was observed in suture samples coated with BGNs+GO than in sutures coated with BGNs. This situation can be explained by the modification of the surfaces of the GO particles during synthesis. In this experimental study, it can be assumed that the strong adhesion observed is due to a mechanical interlocking mechanism between the nanoparticles themselves and the structure suture surface.^30^

The composition of the crystalline particles present on the surface of the BGNs- and BGNs/GO-coated suture samples was performed by FE-SEM-EDX spectra. The spectra collected are presented with the calculated values in Figure 3. As shown in Figure 3, in the case of both coated suture samples, the presence of calcium, phosphorus, silicon, sodium, and oxygen was detected, further confirming the presence of those components in the suture composites. The EDX spectrum for coated samples showed that the layer formed was rich in calcium, phosphorus, silicon, and sodium. Both these elements are present within the glass compositions.^31^ This suggests that either these ions have been incorporated into the BGNs layer or that the thickness of the coated layer was more than uncoated suture samples. The EDX results for S+BGNs and S+BGNs/GO groups showed inclusion peaks of silicon and sodium within the calcium phosphate phase, supporting the difference in morphology seen within the FE-SEM images compared with uncoated suture samples.

**Figure 4:**
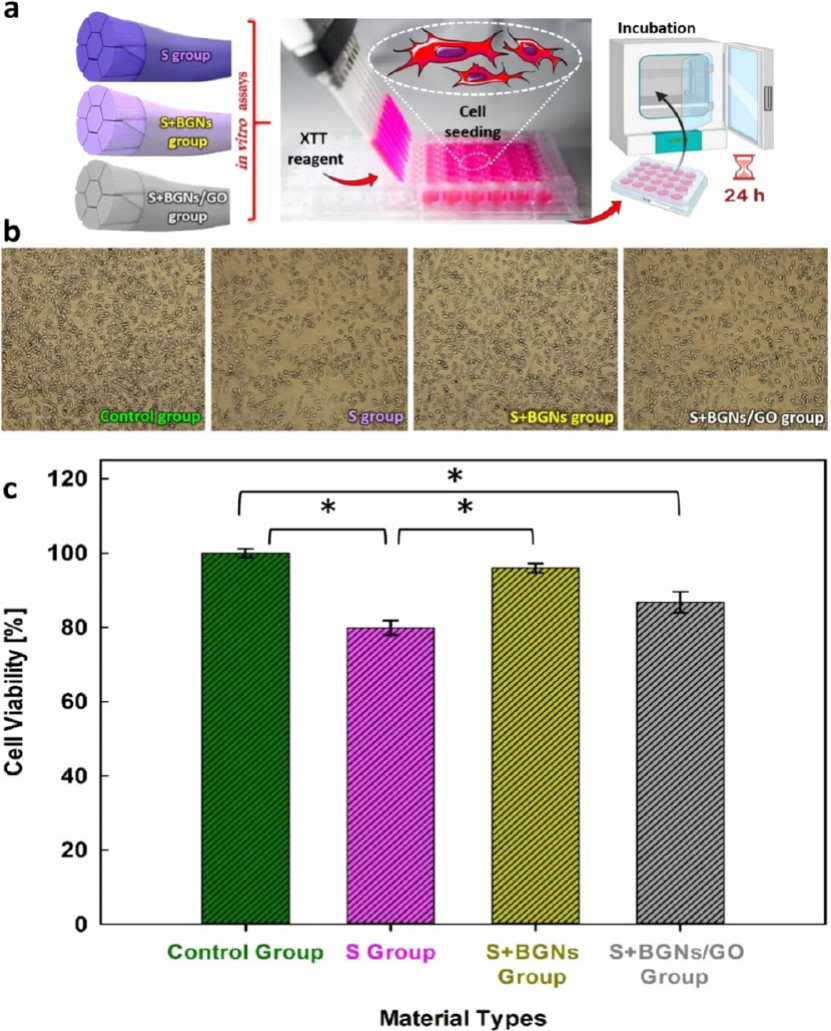
(a) Schematic illustration of the L929 cell-based in vitro assay of suture composite samples in relation to cell viability. (b) In vitro cell viability and 2,3-bis-(2-methoxy-4-nitro-5-sulfophenyl)-2H-tetrazolium-5-carboxanilide (XTT) assay. The observed cell morphology of L929 cells (mouse fibroblast cell line) after being treated for 24 h under a 100 Â inverted microscope. (c) Cell viability diagram (%) by the XTT assay for each group. Values represent the mean and ± SD of three independent experiments (*p* < 0.05; *Statistically significant differences between groups).

Biocompatibility is crucial for suture materials, and the biocompatibility of biomaterials was normally evaluated using cytotoxicity. The cell viability XTT assay was conducted on four groups (control group, S group, S+BGNs group, and S+BGNs/GO group) with cultured L929 in 96-well plates. Each group was incubated for 24 h. Figure 4 shows cell viability photographs of suture samples and the cell viability diagram by the XTT assay. Figure 4 demonstrated that all groups supported cell proliferation with cell viability values higher than 80%. In the experimental study performed with different suture materials, it was observed that both materials were not cytotoxic (Figure 4). The highest cell activity was observed for S+BGNs (95.5 ± 1.23%) and S+BGNs/GO (86.73 ± 2.83%) groups. However, there was a noticeable difference in the cell activity for the S group (80.87 ± 1.93%), which consequently proved beneficial for cell growth. The reason is attributed to the presence of a mixed polycrystalline hydroxyl–carbonate–apatite (HCA) layer on suture surfaces. The dissolution products of the bioactive glass act as cell receptors facilitating cell proliferation and growth.^32^ The results proved that the coated suture samples, compared to the uncoated samples, are highly biocompatible for tissue engineering applications.

The healing functionalities of suture groups with coated BGNs and BGNs+GO were investigated by assessing the degree of skin tissue regeneration and calculating the score classification of the histopathological lesions in a suture-implanted rat model in vivo test.

**Figure 5:**
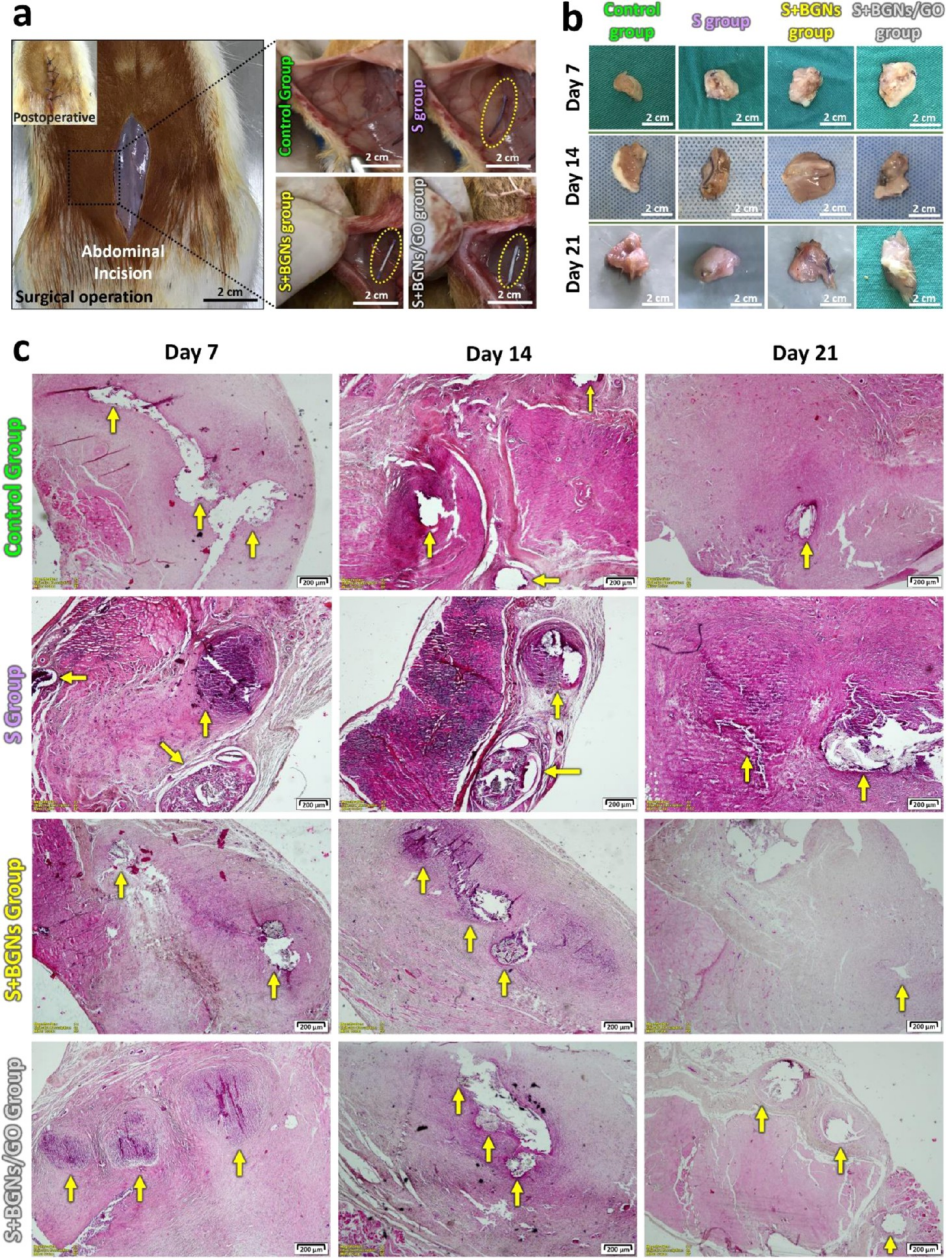
(a) Rat skin macroscopic photographs of the samples of each suture group obtained and the regions applied to the experimental animals during the surgical operation. The yellow circles that are pointed out in the images indicate the locations where the sutures were placed. (b) Appearance of the tissues with fragments taken for histopathological evaluation 7, 14, and 21 days after the surgical operation. In vivo suture samples interacting with the tissue appear to be completely surrounded by the tissue. (c) Photographs of histological analysis (H&E staining, 4× magnification) on H&E-stained cross sections of rat skin from both the epidermis and dermis layers to assess histological changes in rat skin following the surgical procedure.

In surgical applications, biocompatible materials are important to both close the wound and not damage the tissue during the healing process after closure. When the biomaterial is implanted into a tissue, immune system cells are attracted to the implanted biomaterial and attempt to degrade it.^33^ To biodegrade the biomaterial, macrophage-derived cells migrate to the implanted area, and inflammatory reactions may develop in response to phagocytosis by macrophages.^34^ Over time, connective tissue cells migrate to replace the biodegradable material and proliferate, and if the biomaterial is not biodegradable, tight connective tissue capsule formation takes place around the biomaterial, which is considered a foreign material. In addition to these processes, angiogenesis is an indispensable mechanism for cellular proliferation and new tissue regeneration. Each suture group was placed into the peritoneum of the left abdominal sidewall of rats (Figure 5a), and after the healing period, they were sacrificed, and the tissue samples were obtained 7, 14, and 21 days post-implantation. The rats were evaluated macroscopically, and the implanted sutures were removed along with fragments of the surrounding tissue for histopathological evaluation (Figure 5b,c).

In the in vivo tests conducted in this experimental study, the tissue was invigorated and left to heal without any biomaterials placed in the control group. When histopathology results were examined, it was found that in the first 7 days in the control group, the inflammatory cells migrated to the healing area, the damaged area was gradually closed, but the inflammation was not completely lost. When the histopathological photos of the other groups were examined, it was determined that the inflammation was more intense in the S group, starting from the 7th day, compared to the S+BGNs and S+BGNs/GO groups, and the capsule around the biomaterial was thicker. Among all of the groups, the best results in terms of less inflammation, no capsule formation, and the rate of closure of the damaged area in the tissue belong to the S+BGNs group. Inflammation around the biomaterial, characterized mainly by the presence of lymphocytes and macrophages, is accompanied by cell growth of the fibrous tissue between biomaterials, which represents local chronic inflammation and the organization of this tissue.^35,36^ Two weeks after surgery, notably, more polymorphonuclear leukocytes (PMNs) were observed in the rat tissue sample with the S+BGNs and S+BGNs/GO groups than those in the other groups, which indicated that angiogenesis had occurred more efficiently. In particular, this situation is more evident in the S+BGNs group than in the S+BGNs/GO group. We found that intense phagocytosis was significantly higher in the S+BGNs group than in all modified sutures, which was related to fragmentation and biomaterial degradation. This association promoted the phagocytosis of cells by macrophages.^37^ The cell proliferation of the fibrous tissue around implanted sutures may be associated with the presence of apatite formation in the suture surfaces, as observed by FTIR and EDX. The presence of biologically active fragments like Ca^2+^, SiO_4_^4–^, PO_4_^–3^, and Na^+^ ions lead to cell growth, and the mechanism of apatite formation in BGNs is attributed to the accumulation of dissolution products.^38^ Furthermore, in this study, we found that the bioactivity of the S+BGNs/GO group was slightly lower than that of the S+BGNs group, suggesting that GO reduces the bioactivity of BGNs.^39^

**Figure 6:**
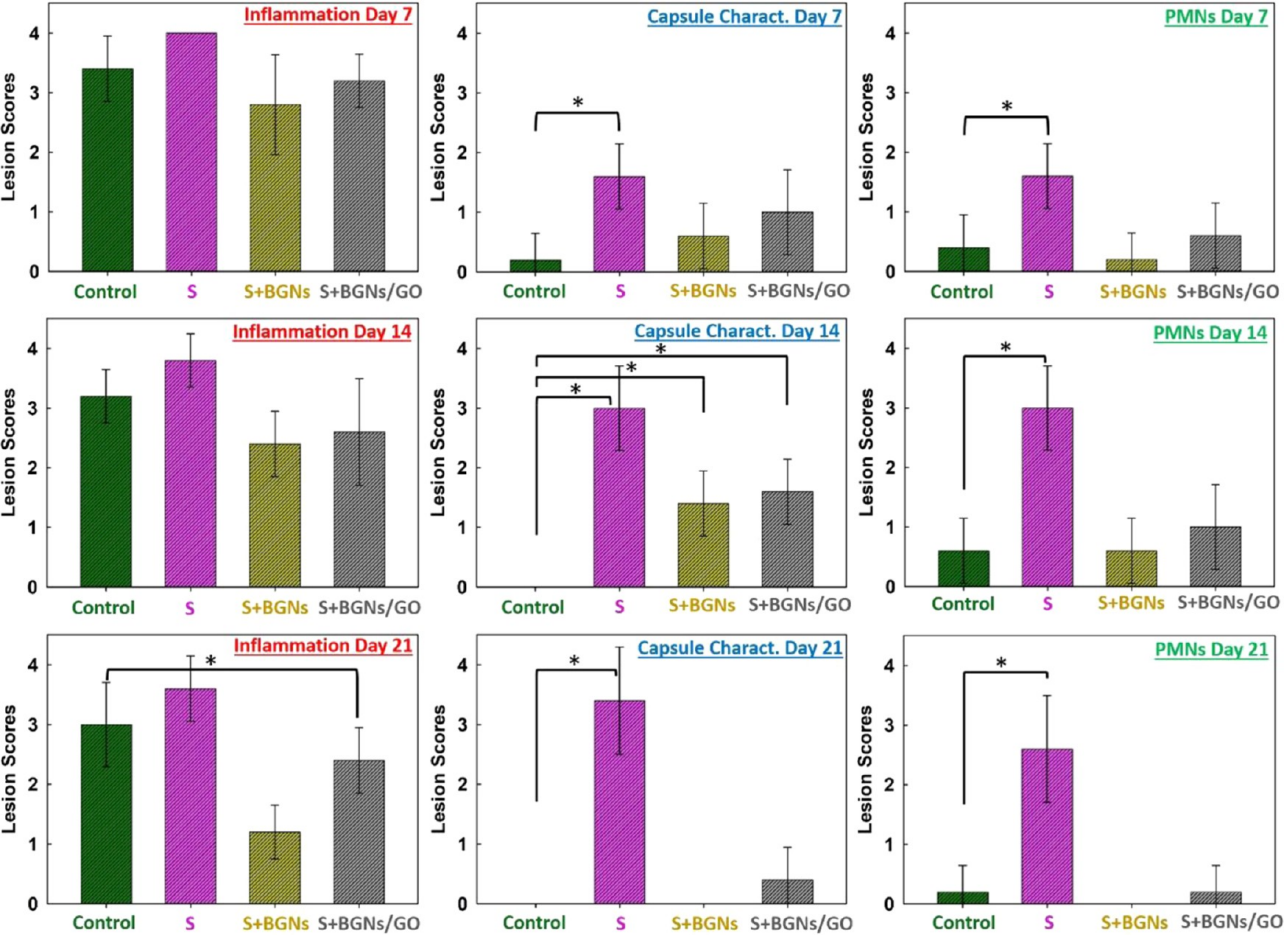
Score classification of the histopathological lesions in the suture groups at each surgical removal time of rats (*n* = 5). Macroscopic evaluation was used to assess inflammation, capsule characterization, and polymorphonuclear leukocytes (PMNs) formation after laparotomy. The intensity of lesions was classified as absent reaction (0), mild reaction (1), moderate reaction (2), marked reaction (3), and severe reaction (4). All data are represented as the mean (standard deviation, ± SD) (*p* < 0.05; *Statistically significant differences between groups).

The inflammation, capsule characterization, and PMNs formed after laparotomy were evaluated macroscopically by two independent researchers unaware of the applications. Grading of fibrotic capsular formation, inflammation, and PMNs was evaluated in terms of histopathological lesion prevalence and severity, with scores from 0 to 4 (Figure 6). Seven days after the surgical operation, inflammatory cells were observed intensely in control, S, S+BGNs, and S+BGNs/GO groups. On the 14th and 21st days, inflammatory cells gradually decreased. This reduction in inflammatory cells was visible, especially in the S+BGNs and S+BGNs/GO groups. It was observed that the fibrotic capsular formation increased in the S group as the day increased from the 7th day through the 21st day, compared to the S+BGNs and S+BGNs/GO groups. The PMNs observed in the S group were even more remarkable compared to S+BGNs and S+BGNs/GO groups. By the first and second weeks of recovery, PMNs increased and then prominently decreased.

**Figure 7:**
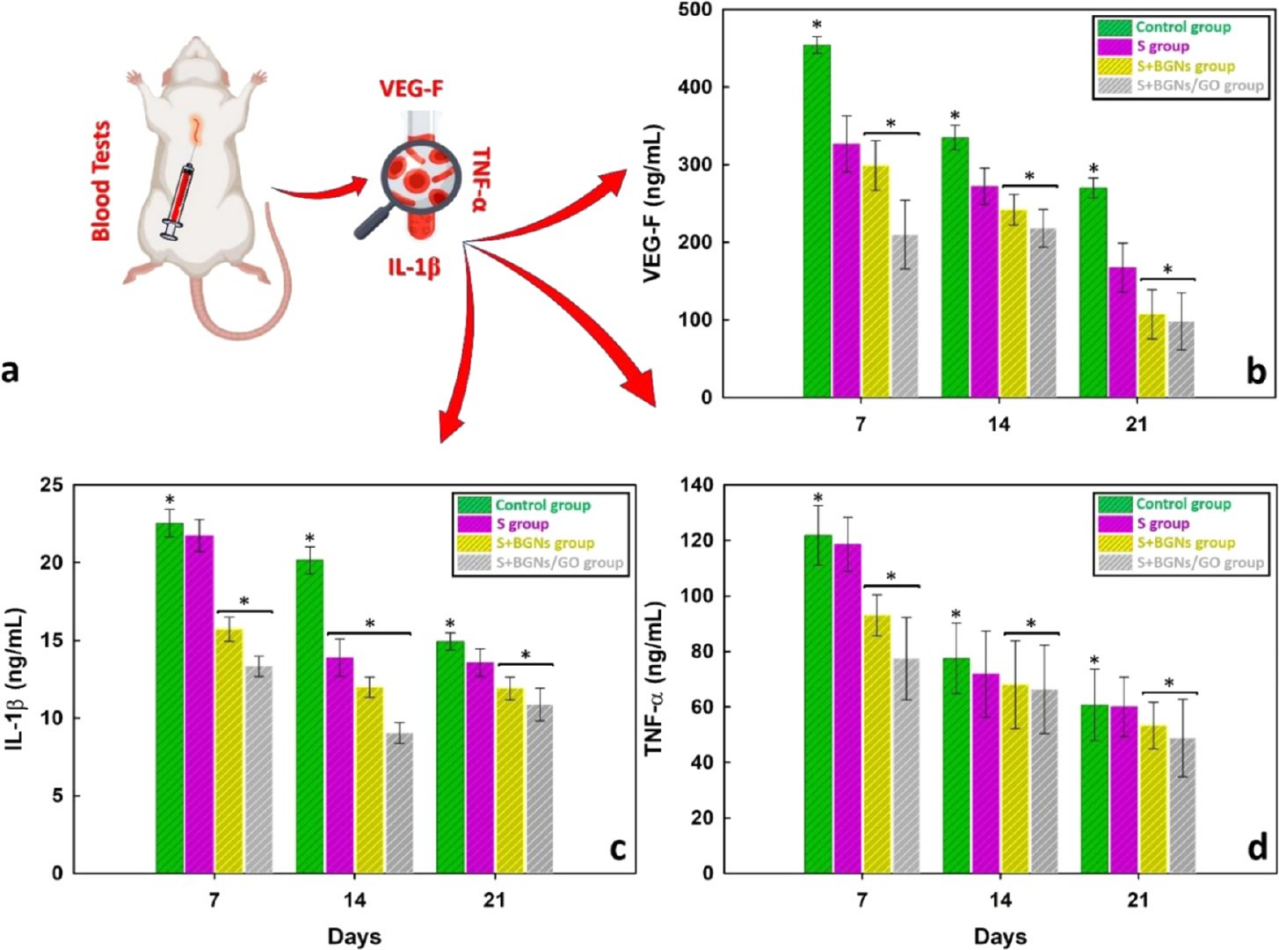
(a) Levels of the vascular endothelial growth factor (VEGF, ng/mL), interleukin-1β (IL-1β, ng/mL), and tumor necrosis factor-α (TNF-α, ng/mL) were determined from the intracardiac blood of rats on days 7, 14, and 21 after surgery. This was done to assess the changes in the levels of these biomarkers over time in each animal group. (b) VEGF levels in each animal group on days 7, 14, and 21 were compared to evaluate any differences in their expression. (c) Similarly, the levels of IL-1β between the groups on days 7, 14, and 21 were compared to assess any variations in their expression levels. (d) Lastly, the TNF-α levels between the groups on days 7, 14, and 21 were compared to determine any differences in their expression levels over time. All of the values are expressed as means (standard deviation, ± SD). Additionally, any statistically significant differences between groups were marked with an asterisk, and the significance level was set at *p* < 0.05.

Biochemical and hematological parameter (VEGF (ng/mL), IL-1β (ng/mL), and TNF-α (ng/mL)) levels in the intracardiac blood taken from animals were measured by the ELISA method (Figure 7a). VEGF was found to be 454.914 ng/mL ± 10.5493 on day 7, 334.1457 ng/mL ± 15.8360 on day 14, and 270.8190 ng/mL ± 12.9458 on day 21 in the control group. In the S+BGNs group, it was found to be 297.3187 ng/mL ± 32.1842 on the 7th day, 242.2640 ng/mL ± 19.5076 on the 14th day, and 107.4413 ng/mL ± 31.5278 on the 21st day. The amount of VEGF decreased with days in the S group and S+BGNs group more in than the other groups (Figure 7b). IL-1β was found in control group to be 22.6017 ng/mL ± 0.7900 at day 7, 20.1959 ng/mL ±0.8200 at day 14, and 14.9626 ng/mL ±0.5380 at day 21, while in the S+BGNs group, it was found to be 15.6143 ng/mL ±0.7900 on day 7, 11.8915 ng/mL ± 0.4500 on day 14, and 11.8915 ng/mL ± 0.8400 on day 21. While the amount of IL-1β decreased as the number of days increased within the groups, the amount of IL-1β decreased even more in the S+BGNs group compared to the control group (Figure 7c). TNF-α was found in the control group to be 121.9249 ng/mL ± 13.5400 on day 7, 77.5294 ng/mL ± 15.6300 on day 14, and 60.6078 ng/mL ± 15.8200 on day 21. In the S+BGNs group, it was found to be 93.2100 ng/mL ± 10.4423 on day 7, 68.1178 ng/mL ± 20.0210 on day 14, and 53.6104 ng/mL ± 10.4156 on day 21. While the amount of TNF-α decreased as the number of days increased within the groups, the amount of TNF-α decreased more in the S group and S+BGNs group compared to the control group (Figure 7d).

## Conclusions

In this paper, the adsorption of BGNs and GO by a surgical suture was achieved by using the VSD method. As investigated and discussed above, bioactive glass/graphene oxide, when placed in soft tissues, allows for inorganic reactions to take place, leading to biochemical and cellular reactions responsible for cell proliferation and thus tissue regeneration. Our experimental data demonstrated that the incorporation of BGNs and BGNs+GO into PGLA sutures greatly enhanced the surface reactivity of the suture surface and promoted the transformation of the polymeric surface into a Ca–Si–P-rich bioactive surface layer. Furthermore, in vitro and in vivo studies of wound healing revealed that the positively charged suture surfaces enhanced the wound healing process via accelerated skin remodeling, which may decrease inflammation formation. The ability of SiO_4_^4–^ ion release during calcium silicate degradation from the suture surface provides biological effects that can be suitable also for the healing of soft tissues. Calcium silicate ions promote the angiogenesis-favoring vascular endothelial growth factor (VEGF) and improve the wound healing process, stimulating the proliferation and migration of cells. Most importantly, these experimental results show that BGNs/GO-coated sutures show promise as a clinically safe biomaterial and should be further evaluated in experimental animal models and human trials. BGNs and BGNs+GO nanoparticles are easy to administer and cost advantageous and could be helpful in surgical practice if their efficacy is confirmed in clinical trials.

## Experimental Section

### Materials

Particle and GO synthesis was performed with analytical grade silicon dioxide (SiO_2_), sodium carbonate (Na_2_CO_3_), calcium carbonate (CaCO_3_), phosphorus pentoxide (P_2_O_5_), potassium permanganate (KMnO_4_), graphite powder (C), sodium nitrate (NaNO_3_), sodium alginate (NaAlg), sodium chloride (NaCl), cetyltrimethylammonium bromide (CTAB, CH_3_(CH_2_)15N(Br)(CH_3_)_3_), 95–98% (w/v) ammonium polyacrylate dispersing agent (DARVAN 821-A), sulfuric acid (H_2_SO_4_), 37% (v/v) hydrochloric acid (HCL), and 30% (w/v) hydrogen peroxide (H_2_O_2_) solution purchased from Sigma-Aldrich (St. Louis). Violet braided resorbable 3/0 Pegelak (PGLA, (poly[glycolide-*co*-l-lactide], (90:10%))) medical sutures were obtained commercially from Doğsan Inc. (İstanbul, Türkiye). The empirical formula of the suture copolymer is −[(C_2_H_2_O_2_)×(C_3_H_4_O_2_)y]n–, where *x*/*y* = 9:1 and the mean diameter of the as-received PGLA suture is 0.3 mm, with individual fibrils of diameters ∼10 to 15 μm. All other in vitro/in vivo experimental supplies and reagents were purchased from Merck KGaA (Darmstadt, Germany), Thermo Fisher Scientific (Massachusetts), and Bayer AG (Leverkusen, Germany).

### Preparation of 45S5 Bioglass Nanoparticles (BGNs) and Synthesis of Graphene Oxide (GO) Nanosheets

**Figure 8:**
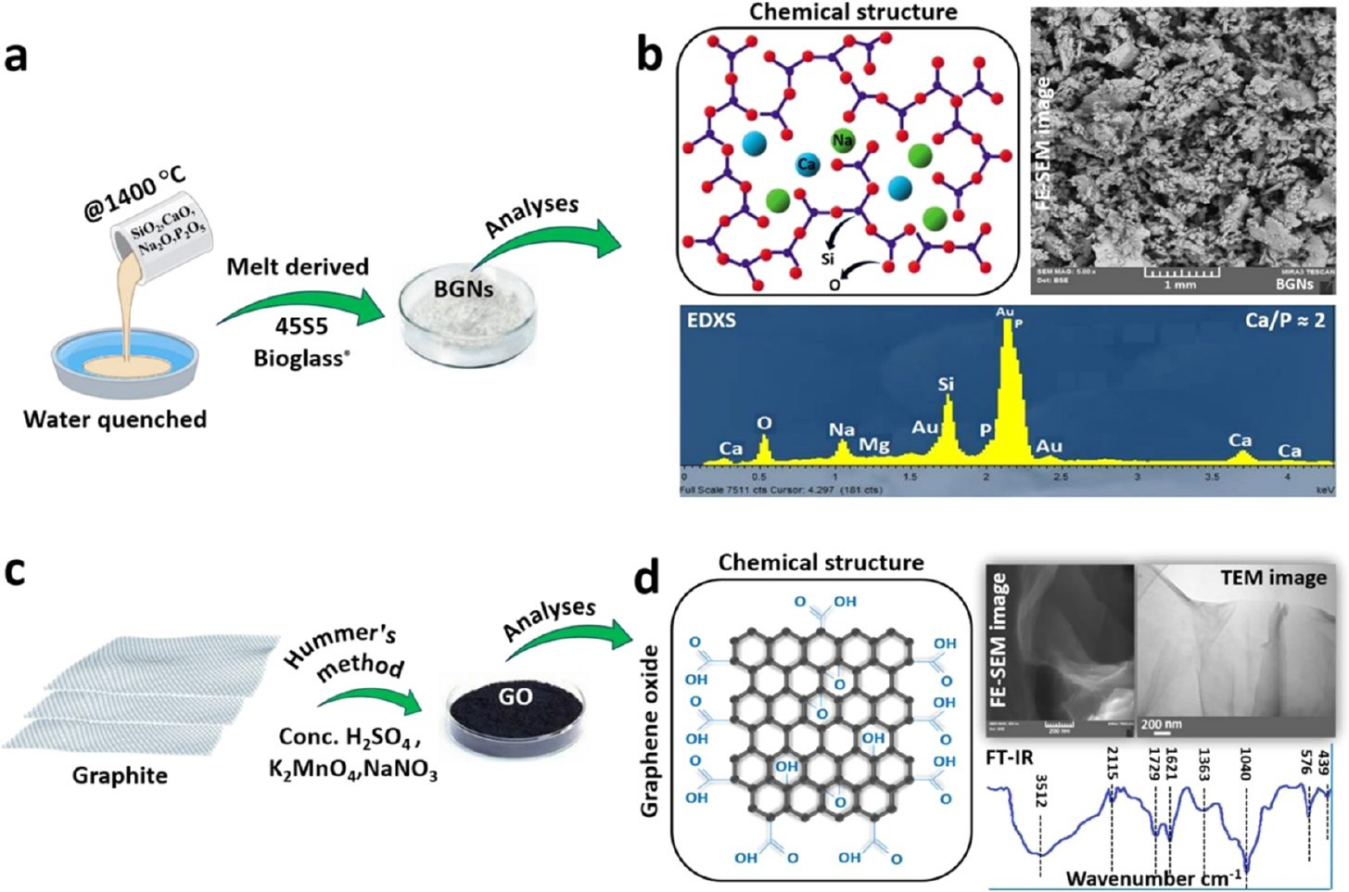
Preparation of high-performance 45S5 bioglass nanoparticles (BGNs) and synthesis of graphene oxide (GO) nanosheets. (a) Schematic demonstrating the preparation of the melt-derived BGNs. The process involves the melting of the 45S5 bioglass material at high temperatures, followed by rapid quenching to produce a glassy matrix. This matrix is then ground into fine particles to produce the BGNs. (b) Chemical structure of BGNs: The BGNs are composed of various elements such as Si, Na, Ca, and P, which are essential for tissue repair. The top-view FE-SEM images of the BGNs show the morphology of the nanoparticles, with a uniform size distribution and an irregular shape. The EDX spectra of the BGNs confirm the presence of the related elements in the composition of the nanoparticles. (c) Experimental demonstration of the synthesis process of the GO by Hummer’s method. This method involves the oxidation of graphite using a mixture of concentrated H_2_SO_4_, K_2_MnO_4_, and NaNO_3_. (d) Resulting GO material is composed of carbon, hydrogen, and oxygen, as demonstrated by the formation of its molecular structure. The FE-SEM image shows the morphology of the GO material, with a thin and flat structure that forms a nanosheet. The magnified TEM image provides a closer look at the nanosheet, revealing its layered nanostructure. The FTIR spectra of the GO material show the presence of various types of oxygen functional groups (C**–**OH, C**–**O**–**C, C=O, and O=C**–**OH), which are characteristic of GO.

High-purity SiO_2_, Na_2_CO_3_, CaCO_3_, and P_2_O_5_ powders were weighed and mixed to obtain 45S5 bioactive glass (45 SiO_2_, 24.5 CaO, 24.5 Na_2_O, 6 P_2_O_5_ in (wt % ) melt-derived method). The raw powders were mixed for 6 h using a T2F turbula mixer and then melted in a platinum crucible (95% Pt–5% Au) for 4 h at 1400 °C with a decarbonization step (5 h at 950 °C). The melted 45S5 bioglass was then quenched into distilled water (dH_2_O). The frit glass was then milled in a planetary ball mill in C_2_H_5_OH to a nanopowder (≤100 nm).^40,41^ The bioglass composition was checked by chemical and spectral analysis to ascertain that no impurity was present after the preparation steps (Figure 8a,b).

GO was synthesized using the modified Hummer’s method from pure graphite powder. The details of the modified Hummer’s method are well described in ref (42). Briefly, 40 mL of H_2_SO_4_ was stirred in an ice bath (0 °C) for about 15 min. Graphite powder (≈2 g) was placed into the H_2_SO_4_ solution under stirring conditions. KMnO_4_ and NaNO_3_ were then added slowly into the solution by keeping the temperature below 10 °C. This mixture was stirred for 6 h and then allowed to react at 35–40 °C in a water bath for 24 h. 70 mL of dH_2_O was added and stirred until the solution became brown. This was followed up by another addition of 110 mL of dH_2_O for further dilution. The suspension was further treated by adding a mixture of H_2_O_2_ and dH_2_O water to convert the residual permanganate and MnO_2_ into soluble MnSO_4_. This treatment turned the solution bright yellow. The remaining mixture was centrifuged and washed 3 times using 5% HCl in dH_2_O to cleanse any residue. After drying in an oven at 90 °C for 24 h, a solid sample of GO could be obtained (Figure 8c,d).

### Sol Preparation and Gelation

**Figure 9:**
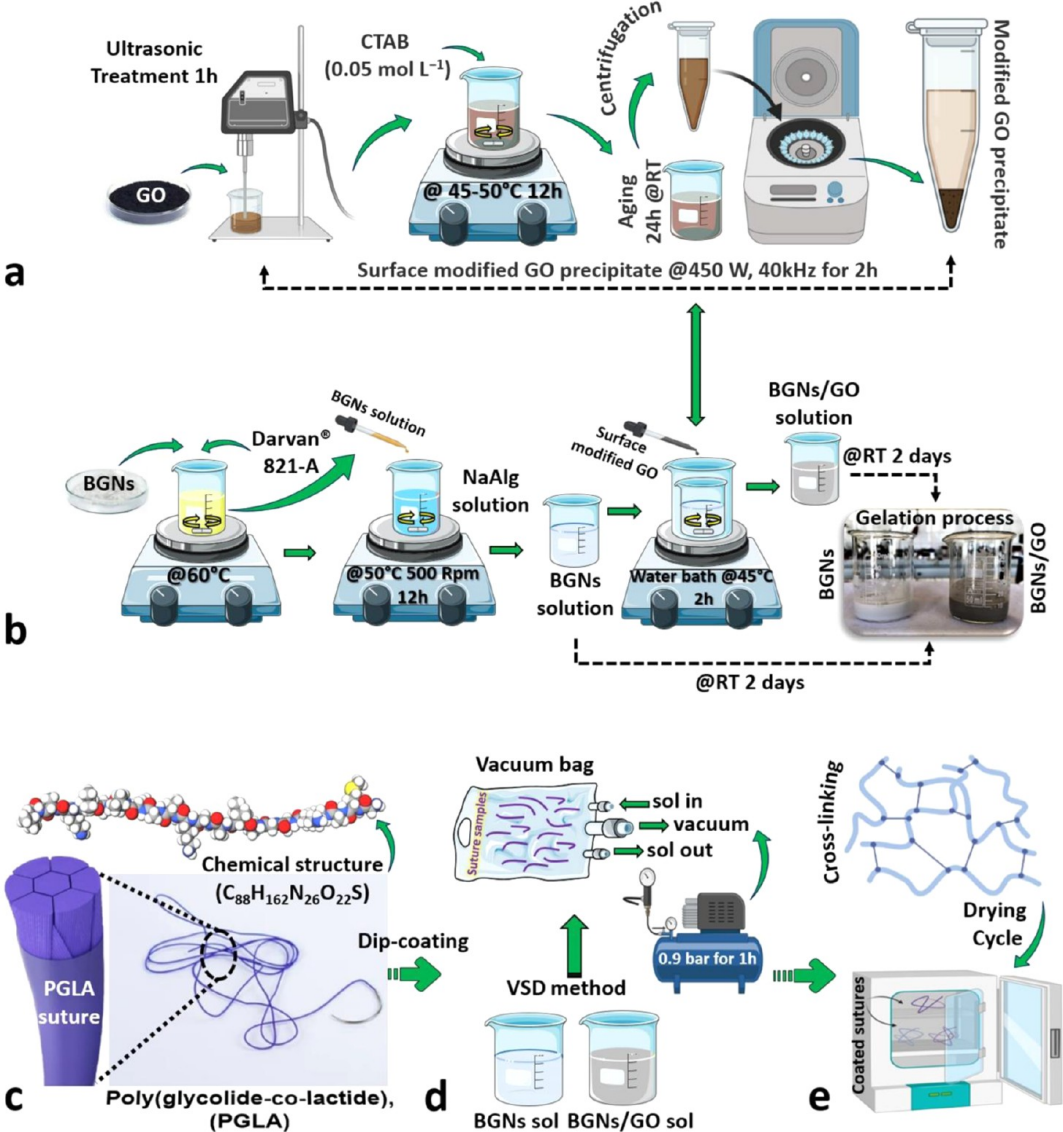
Layout of the sol/gelation methodologies and vacuum sol deposition (VSD) process. (a) Surface-modified GO precipitate is subjected to ultrasonic waves at a frequency of 40 kHz and a power of 450 W for a period of 2 h. This process enhances the homogeneity of the GO dispersion and increases its surface area, making it more suitable for use in subsequent processing steps. (b) Desired volume of sols can be produced by mixing BGNs and BGNs/GO with a liquid solvent, which facilitates the formation of a sol. The sol preparation process involves mixing, stirring, and sonication to ensure a homogeneous mixture. (c) Chemical structure of the PGLA (poly(glycolide-*co*-lactide)) suture. The chemical structure of the PGLA suture is a long-chain polymer composed of repeating units of glycolide and lactide. (d) Experimental demonstration of the coating process of the suture composite samples by the VSD method. This process involves preparing a liquid sol of the coating material and depositing it onto the suture composite samples using a vacuum chamber. (e) Drying and cross-linking cycle of coated suture composite samples (20 cm length) is described in this step. Once the coating has been applied, the samples undergo a drying and cross-linking cycle to ensure that the coating adheres to the surface of the suture composite samples. The cross-linking process involves the formation of chemical bonds between the coating material and the surface of the suture composite samples, resulting in a strong and durable bond.

The suture composites were prepared with a vacuum-assisted sol deposition (VSD) technique, similar to that reported earlier, to coat surgical meshes with 45S5 bioglass particles.^43,44^ Quantitative GO was ultrasonically dispersed into C_2_H_5_OH for 1 h in a bath sonicator to distribute GO evenly throughout the aqueous solution. After that time, the bath temperature was heated to 45–50 °C, and a given amount of CTAB (0.05 mol L^–1^, CTAB/GO = 25 mmol/g) was added into the GO suspension under magnetic stirring and kept at the temperature for 12 h with stirring and aged for 24 h at RT. After centrifugation, surface-modified GO precipitate was obtained. 0.75 g of BGNs was dispersed into 1 mL of dH_2_O, and 0.25 g of NaAlg was dissolved into 8 mL of dH_2_O at 60 °C. Then, the BGN suspension and an optimized quantity of ammonium polyacrylate solution as a dispersing agent (DARVAN 821-A) were dropped into the NaAlg solution and subsequently stirred at 500 rpm for 12 h to prevent particle agglomeration. The obtained surface-modified GO precipitate (0.05 g) was dispersed into 1 mL of dH_2_O via ultrasonic treatment (450 W, 40 kHz) for 2 h to obtain a suspension. Then, the prepared GO solution was added drop by drop into the BGNs suspension in a water bath at 45 °C, and the suspension was constantly stirred for 2 h. To allow the hydrolysis and polycondensation reactions to occur, the prepared solutions were kept sealed at RT for 2 days until the gelation process was complete. The same experimental procedure was applied to the undoped GO sol preparation process. Figure 9a,b is a schematic representation of the BGNs and BGNs/GO sol/gelation method.

### Processing of BGNs and BGNs/GO Suture Samples

A vacuum sol deposition method with multiple cycles was devised to process BGNs- and BGNs/GO-coated suture composites. Sutures were cut into equal lengths of 25 mm and placed over an aluminum plate to prepare the setup for deposition. Afterward, a polyester peel ply, a distribution mesh, and resin in-out pipes were attached and adjusted accordingly. An airtight nylon vacuum bag was placed over the setup, and a vacuum pump was attached to generate a constant vacuum pressure of 9 × 10^–1^ bar for 1 h. The inflow of BGNs and BGNs/GO sol was kept at a slow rate in order to achieve complete wetting of the multifilament suture preform. Subsequently, the vacuum pump was switched off while keeping the setup under vacuum. BGNs-coated suture composites and BGNs/GO-coated suture composites were removed by the ends, maintaining their orientation, and withdrawn carefully to prevent damage at a withdrawal velocity of ∼10 mm s^–1^. The coated sutures were then dipped sequentially for 5 min into a 0.1 M NaCl solution, followed by a 10-minute rinse in warm dH_2_O. The coated sutures were dried on glass plates at ambient temperature in a humid atmosphere to minimize the development of microcracks in the BGNs and BGNs/GO coating. The obtained suture samples are denoted as the S group, BGNs group, and BGNs/GO group, correspondingly. For comparison, the pure commercial suture was prepared in the above similar manner without the addition of BGNs and GO. The slurry concentrations, immersion times, and the level of pressure applied were optimized by a trial-and-error approach to producing as uniform a coating as possible. The optimization was based mainly on the visual and field emission scanning electron microscopy (FE-SEM) observation of BGNs and BGNs/GO particles attached mechanically to the surface of the sutures. Five experimental steps were followed to treat the surgical sutures with the coating, namely, pretreatment (cleaning), dip coating, VSD method, drying, and cross-linking, as shown in Figure 9c–e.

### Materials Characterization

As-received and coated suture samples were characterized using field emission scanning electron microscopy (FE-SEM, Tescan Mira3 XMU, Brno, Czechia) to study the fiber thickness, morphology, homogeneity, and structure of the coatings. Suture samples were sputter-coated in gold for 2 min at 20 mA and observed at an accelerating voltage of 10–20 kV. Composition analysis of suture composite samples was performed using an energy-dispersive spectroscopy apparatus attached to a scanning electron microscope (EDX, INCA IE 350, and U.K.). The surface chemical signatures of the different coated suture composites were assessed with FTIR spectroscopy (Bruker α II FTIR Spectrometer, Germany). The spectral range detected was 400–4000 cm^–1^ with a wavenumber resolution of 2 cm^–1^. Using the ATR function, the suture fiber chemical bond vibrations were characterized using infrared light and tabulated using OPUS spectroscopic software.

### In Vitro Cytocompatibility

L929 mouse fibroblast cells (American Type Culture Collection, ATCC) were selected as a model cell line for the cytocompatibility assay and maintained in Dulbecco’s modified Eagle medium (DMEM) supplemented with 1% (v/v) of a premade penicillin (100 units/mL) and streptomycin (100 units/mL) solution and 10% (v/v) fetal bovine serum (FBS). The culture medium was replaced after every 2–3 days, and cells were grown at 37 °C in a humidified incubator in 5% CO_2_. The cytotoxic activity was determined using the 2,3-bis-(2-methoxy-4-nitro-5-sulfophenyl)-2H-tetrazolium-5-carboxanilide (XTT) technique. L929 mouse fibroblast cells were seeded on a 96-well plate with growth media, then treated with various compositions of suture samples, and incubated for 24 h in a humidified CO_2_ atmosphere at 37 °C. Following incubation, the XTT assays were performed, which required 10 μL of XTT reagent to be added to each well and 2 h of incubation at 37 °C and 5% CO_2_. The absorbance was measured spectrophotometrically with a Thermo Scientific Multiskan FC Microplate Photometer reader (Thermo Scientific) at a wavelength of 475 nm. The viability of the cells was calculated as a percentage of the viability in the control test, which was considered 100%.^45^

### Animal Model Studies In Vivo

All surgical procedures and perioperative care measures were conducted in compliance with the Sivas Cumhuriyet University Institutional Animal Care and in accordance with The Guide for the Care and Use of Laboratory Animals of the National Institutes of Health. In the experimental study, 60 Wistar albino rats, newly adult, 16 weeks old, unmated, female, 200–220 g, were used as experimental animals. After the rats were purchased, they were kept for one week to acclimate to the laboratory conditions. Rats were kept as two individuals in a cage with standard animal housing conditions in the laboratory, 12 h of light–darkness, 21 °C temperature, 50–60% humidity, and standard pellet rat chow and water.^46^ A total of 60 rats were assigned randomly to four independent groups: the control group, S group, S+BGNs group, and S+BGNs/GO group. Each group was divided into 7th-, 14th-, and 21st-day groups among themselves. There were 5 rats in the subgroups. All rats fasted for 12 h before surgery. Anesthesia was administered through intraperitoneal injection of 10 mg/kg Xylazine and 50 mg/kg Ketamine, and spontaneous breathing was achieved. The rats were fixed in the supine position, and the midline lower abdomen of each rat was shaved, cleaned, and disinfected with a 2% iodine solution prior to the procedure. An approximately 3 cm midline vertical skin incision was made and reached the peritoneum. Prior to the implantation, these tested suture samples were transferred to the UV sterilization unit for 5 min. Prepared suture samples were used right after UV sterilization and placed between the peritoneum and subcutaneous tissue to evaluate the in vivo biocompatibility. The surgery was terminated by closing the skin. The rats were housed in ventilated rooms and allowed to eat and drink *ad libitum* after surgery. The animals were sacrificed 7, 14, and 21 days after implantation. No antibiotic prophylaxes were administered, and no mortality occurred during the experiment. All surgical procedures were performed by the same surgeon under the same experimental condition.

### Evaluation of Biochemical and Hematological Parameters

Evaluation of biochemical and hematological parameters was performed in the 7th-, 14th-, and 21st-day groups. After the animals were sacrificed, 5cc of blood was taken into a tube with EDTA by the intracardiac route.^47^ The collected fresh blood was centrifuged at 3000 rpm for 15 min and then separated into the serum. TNF-α (tumor necrosis factor α, Cat No: E0764Ra) and IL-1β (interleukin-1β, Cat No: E010Ra) from cytokines and VEGF (vascular endothelial growth factor, Cat No: E065Ra) from the growth factor was studied in blood serum samples taken following the guide of the kit for the ELISA method.

### Histopathological Studies and Biological Evolution

Histopathological studies and biological evolution were performed in the 7th-, 14th-, and 21st-day groups. The rats were sacrificed, and regrown hair was removed. The wounds were excised, along with an area of normal skin 5 mm around the wound, after which the excised tissues were pinned flat on dental wax. Tissue fragments were fixed in 10% neutral buffered formalin at 4 °C for 48 h. After fixation, the samples were washed in dH_2_O, dehydrated in an increasing series of ethyl alcohol, cleared in xylol, and embedded in paraffin blocks after the tissue follow-up processes according to the standard protocol. 3–5 μm thick sections were cut with a Leica RM 2245 microtome using disposable blades, and immediately, routine hematoxylin–eosin (H&E) staining was performed to evaluate the layers of the tissue and the healing rates in the wound.

For immunohistochemical evaluation, tissue blocks were resectioned, deparaffinized, passed through a decreasing series of ethyl alcohol, and washed in buffered phosphate solution (PBS) for 5 min. At the end of the period, the sections were boiled in buffered citrate at pH 6 for 20 min in the microwave for antigen retrieval. Sections cooled at RT were washed in PBS for 5 min and kept in a 3% H_2_O_2_ solution prepared with distilled water for 10 min to suppress endogenous peroxidase activity. At the end of the time, the sections were washed with PBS for 3–5 min and then kept at RT for 20 min by dripping ultra V block to prevent nonspecific binding. With the help of blotting paper, the block solution was removed without washing, and the primary antibodies VEGF (monoclonal antibody, JH121) and TGF-β1 were kept in a humid and dark environment for 1.5 h at 37 °C. After the primary antibody application, the sections were washed with PBS for 3–5 min, and the first biotinylated goat anti polyvalent was dripped, washed with PBS, and then streptavidin peroxidase was dropped and kept in a humid and dark environment at 37 °C for 20 min. At the end of the time, the sections were washed with PBS, and chromogen was dripped onto 3,3′diaminobenzidine (DAB) and waited for 5 min. Colored sections were washed with PBS, treated with Mayer’s hematoxylin solution for 1 minute, ground-dyed, rewashed, and covered with a particular concealer. The stained sections were examined with an Olympus BX51 (Japan) microscope, and images were captured and analyzed with the software Image Pro Lite.^46^ Moreover, tissue samples were analyzed for inflammation, capsule characterization, and polymorphnuclear leukocyte (PMN) pathology by two pathologists with no knowledge about the biomaterial group removal time, thus eliminating bias. Tissue reactions to the implanted suture groups were scored semiquantitatively according to the following criteria: (0) absent reaction; (1) mild reaction; (2) moderate reaction; (3) marked reaction; and (4) severe reaction.^36,46^

### Statistical Analyses

The collected data were presented as means ± standard deviations of the mean SD based on at least three independent measurements. All data were statistically analyzed using one-way ANOVA, followed by the Tukey and Holm–Šídák test, and significance was achieved at *p ≤ 0.05 using Origin Pro 9.0 software.

## Supporting Information Available

Movie S1: Clinical application of suture materials on animals (AVI)

## Supplementary Material

ao3c00978_si_001.avi
